# Solubility of Foreign Molecules in Stratum Corneum Brick and Mortar Structure

**DOI:** 10.1021/acs.langmuir.2c03092

**Published:** 2023-01-30

**Authors:** Quoc Dat Pham, Bruno Biatry, Sébastien Grégoire, Daniel Topgaard, Emma Sparr

**Affiliations:** † Division of Physical Chemistry, Chemistry Department, Lund University, P.O. Box 124, 22100Lund, Sweden; ‡ Gillette Reading Innovation Centre, 460 Basingstoke Road, ReadingRG2 0QE, Berkshire, U.K.; § L’Oréal Research & Innovation, 1, avenue Eugène Schueller, 93601Aulnay sous Bois, France

## Abstract

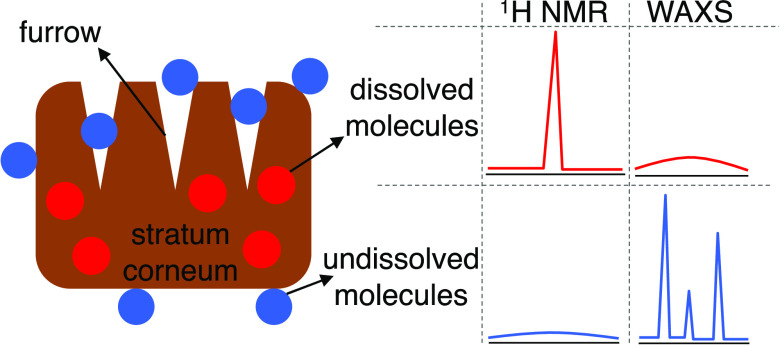

The barrier function of the skin is mainly assured by its outermost layer, stratum corneum (SC). One key aspect in predicting dermal drug delivery and in safety assessment of skin exposure to chemicals is the need to determine the amount of chemical that is taken up into the SC. We here present a strategy that allows for direct measures of the amount of various solid chemicals that can be dissolved in the SC in any environmental relative humidity (RH). A main advantage of the presented method is that it distinguishes between molecules that are dissolved within the SC and molecules that are not dissolved but might be present at, for example, the skin surface. In addition, the method allows for studies of uptake of hydrophobic chemicals without the need to use organic solvents. The strategy relies on the differences in the molecular properties of the added molecules in the dissolved and the excess states, employing detection methods that act as a dynamic filter to spot only one of the fractions, either the dissolved molecules or the excess solid molecules. By measuring the solubility in SC and delipidized SC at the same RHs, the same method can be used to estimate the distribution of the added chemical between the extracellular lipids and corneocytes at different hydration conditions. The solubility in porcine SC is shown to vary with hydration, which has implications for the molecular uptake and transport across the skin. The findings highlight the importance of assessing the chemical uptake at hydration conditions relevant to the specific applications. The methodology presented in this study can also be generalized to study the solubility and partitioning of chemicals in other heterogeneous materials with complex composition and structure.

## 1 Introduction

The outermost layer of the epidermis, the stratum corneum (SC), is composed of layers of corneocytes embedded in a lipid matrix with lamellar arrangement.^1^ The unique composition and structural arrangement of SC components make it an efficient barrier toward diffusional transport of any chemicals from the surrounding.^2^ The barrier function is thus essential for protecting the body against hazardous chemicals as well as extensive water loss. However, there are situations when one aims to overcome the SC barrier, for example, in applications of (trans)dermal drug delivery. To evaluate the absorption and transport in the SC, it is crucial to know the overall solubility of the chemicals of interest in the complex and heterogeneous SC material. In addition, to get insight into the distribution between different regions of the SC as well as what the transport route across the SC is, one needs to know how the added chemical distributes between the extracellular lipids and the corneocytes.

**Figure 1:**
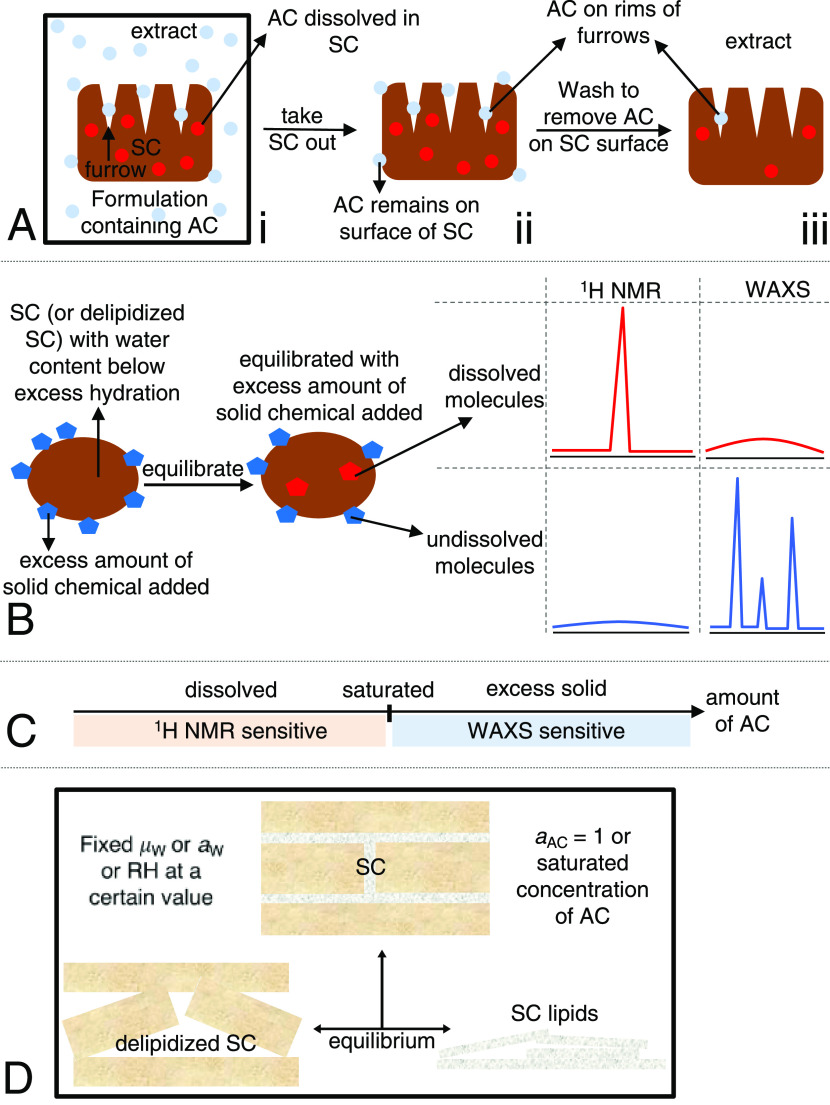
(A) Schematic illustration of possible issues when measuring SC penetration of added chemical (AC); (B) sample preparation procedure and strategy to distinguish molecules that are dissolved in SC (delipidized SC) from molecules that are not incorporated in SC (delipidized SC) by intensity and lineshape of NMR and WAXS signals of molecules in dissolved and undissolved states; (C) overview of the sensitivity of the methods in the different concentration regimes of added chemical; (D) schematic illustration of how chemical potentials of water (μ_w_) and added chemical are controlled to be the same in SC, delipidized SC, and SC lipids; *a*_w_ and *a*_AC_ are activities of water and added chemical, respectively.

One common approach to measure the solubility of chemicals in SC is to study their partitioning between SC and an excess aqueous solution or a formulation.^3−5^ A fundamental complication with these methods is that besides the uptake of various chemicals from the formulation into SC, there will also be extraction of molecules from the SC into the external solution (Figure 1Ai). The extracted chemicals can be small polar molecules as part of the natural moisturizing factor (NMF) if the SC is placed in contact with an excess aqueous solution,^6^ or hydrophobic compounds like fatty acids if the SC is instead exposed to a more hydrophobic formulation/solvent.^7^ The extraction of any SC compound will alter the SC composition, which likely also influences its molecular organization,^6^ and thus also its ability to dissolve the added chemicals. Another experimental difficulty when measuring the solubility of added chemicals in SC relates to the need to distinguish the molecules that are actually taken up into SC from molecules that are deposited on the surface of the SC pieces, including molecules that are on the rims of the furrows (Figure 1Aii–iii).^8^ With the perspective of these complications, we emphasize a need for a method that can distinguish between the dissolved and the excess of the added chemical, and at the same time avoid extraction of SC components to the added formulation or uptake of other chemicals than the molecules of interest.

One important aspect to consider when characterizing solubility in SC is the hydration condition. Most of the existing protocols used to measure the solubility of chemicals in SC are performed in conditions where the SC is placed in contact with an excess solution that contains the molecule of interest.^4^ If this is a dilute aqueous solution, the SC is then close to fully hydrated. However, in many relevant situations, the skin is rather exposed to a drier environment. Variation in skin hydration is known to influence the skin properties, including the fluidity of the SC components,^9−12^ and it likely also impacts the solubility of the foreign hydrophilic or hydrophobic compounds inside the SC. For most cases, the added chemical will have higher solubility in fluid regions of SC compared to solid regions. These fluid regions can be either the small fluid fraction of the extracellular lipids^13^ or the water-rich corneocytes at high skin hydration. Increasing hydration will not only lead to a higher amount of water in these regions but also shift the balance between solid and fluid SC lipid and protein components.^14,15^ The dissolution and partitioning of the added chemicals may therefore change with the hydration of SC. Furthermore, the dissociation of functional groups, e.g., carboxylic acids, can be altered by changes in water activity, which in turn will determine the proportion between the charged and uncharged states of the added chemical.^16,17^ In summary, the solubility of foreign chemicals in the SC shows a strong dependence on the hydration conditions, which in turn depend on the relative humidity (RH) of the environment. This illustrates the need for methodologies that enable the quantification of solubility of various chemicals inside SC under ambient conditions and not only at full hydration or in exposure to other solvents.

**Figure 2:**
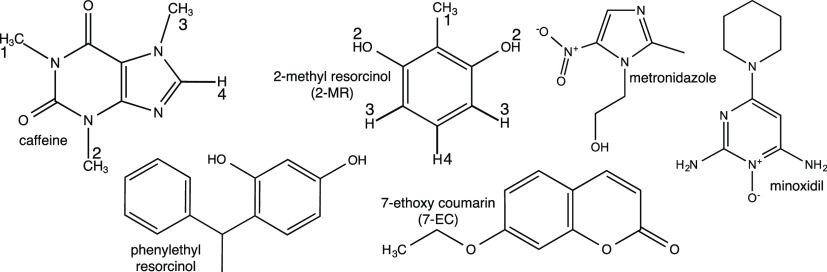
Chemical structures of different added chemicals investigated in this study. Numbered hydrogens of caffeine and 2-methyl resorcinol for assigning their ^1^H NMR spectra are also shown.

In this paper, we present a novel methodology to measure the solubility of chemicals in SC that overcomes all complications described above. The same method is also applied to samples composed of SC where the extracellular lipids have been removed through extraction in organic solvents. The latter sample is herein referred to as delipidized SC and consists of interconnected corneocytes. The method relies on strict control of the thermodynamic conditions, together with the use of experimental tools that distinguish molecules that are dissolved in SC from the excess undissolved molecules. We also discuss how the same methodology can be employed to measure the distribution of the added chemical between the extracellular lipids and the corneocytes within the SC. The method can be used for ambient hydration condition and for solid hydrophobic chemicals without the use of organic solvents that also alter the SC properties.^7,18,19^ We here demonstrate the use of the method in studies of the solubility of a range of chemicals representing different molecular classes with respect to structure and hydrophobicity (Figure 2), all of them are relevant to (trans)dermal pharmaceutical, cosmetic, and sanitary applications. Finally, we also point out that the methodological approach is general and can also be used to study solubility and partitioning of chemicals in other heterogeneous materials with complex composition and structure.

## 2 Materials and Methods

### 2.1 Materials

NaCl, Na_2_HPO_4_·2H_2_O, KH_2_PO_4_, KNO_3_, K_2_SO_4_, trypsin, D_2_O (99.9 atom % D), 2-methyl resorcinol (2-MR), and 7-ethoxycoumarin were purchased from Sigma-Aldrich. Metronidazole was from Duchefa Biochemie (Netherlands). Caffeine, phenylethyl resorcinol, and minoxidil were obtained from L’Oreal (France). Phosphate-buffered saline (PBS) contained 130.9 mM NaCl, 5.1 mM Na_2_HPO_4_, 1.5 mM KH_2_PO_4_, pH 7.4. Milli-Q water (Milli-Q, Merck) was used to prepare PBS and saturated solutions of caffeine and 2-MR.

### 2.2 Preparation of Dermal Skin, Stratum Corneum, and Delipidized SC

Porcine ears were obtained from a local abattoir as byproduct of food production and stored at −80 °C until use. Hair was removed by a trimmer, and the skin from the inner ear was dermatomed (TCM 3000 BL, Nouvag, Switzerland) to a thickness of approximately 500 μm. To separate SC from tissue, the dermatomed skin strips were placed on filter paper soaked in PBS solution with 0.2 wt % trypsin at 4 °C overnight. Sheets of SC were removed by forceps, washed with PBS five times, dried under vacuum, and stored in a freezer until further use.

To prepare SC powder, dry SC was pulverized using a pestle and mortar, dried again in vacuum, and stored in a freezer until further use. To reduce complications related to the biological variation between the individuals, a batch consisting of pulverized SC from different individuals (ca. 40 pig ears) was prepared, and all internal comparisons were performed with samples from the very same batch of pulverized SC for all NMR (nuclear magnetic resonance) and WAXS (wide-angle X-ray scattering) experiments. It is noted from comparisons between previous NMR studies on SC^13,20^ using batches of smaller size (ca. 15 pig ears) that ^13^C NMR signals and molecular mobility of SC components are similar between the batches, confirming that the batch-to-batch variation is remarkably small. Additional WAXS experiments were performed on samples composed of SC sheets and 2-MR prepared and equilibrated in the same way as pulverized SC samples (Figure S1A), showing no significant differences in the measured solubility in the SC sheets and pulverized SC (compare results in Figure S1A and Table 1). Previous studies^15^ also compared SC molecular dynamics in SC sheets and pulverized SC, showing no detectable differences on the length scales studied here.

**Table 1:** Solubility of Caffeine and 2-Methyl Resorcinol (2-MR) in SC or Delipidized SC (dSC) Controlled at Different RHs (%) Using D_2_O^a^

	log *P*_O/W_	*S*_W_	*T*_m_	*M*_w_	system	RH	*W*_SC(dSC)_	*m*_AC-SC(dSC)-sat_/*m*_SC(dSC)_	*F*_SC(dSC),sat_	*S*_SC(dSC)_	*m*_AC-SC(dSC)-sat_/*m*_W-SC(dSC)_	*m*_AC-sat.sol_/*m*_W-sat.sol_
caffeine	–0.07^30^	26^b^	238^30^	194	SC	97	50 [47]	0.050 ± 0.0003	4.7 ± 0.03	2.4 ± 0.02	0.050 ± 0.0003	0.02
								0.03–0.04^c^	3–4^c^	1.5–2.0^c^	0.031–0.042^c^	
						93	38 [36]	0.030 ± 0.001	3.0 ± 0.08	1.9 ± 0.05	0.050 ± 0.001	
					dSC	97	43 [40]	0.056 ± 0.001	5.3 ± 0.05	3.1 ± 0.03	0.074 ± 0.001	
						93	28 [26]	0.02–0.03^c^	2–3^c^	1.4–2.2^c^	0.052–0.080^c^	
2-MR	1.6^30^	321^b^	120^30^	124	SC	97	52 [49]	0.67 ± 0.001	40 ± 0.05	24 ± 0.04	0.62 ± 0.001	0.43
								0.60–0.67^c^	37.5–40^c^	22–24^c^	0.55–0.62^c^	
						93	38 [36]	0.48 ± 0.002	32 ± 0.1	23 ± 0.08	0.78 ± 0.004	
					dSC	97	43 [40]	0.60 ± 0.001	37 ± 0.05	25 ± 0.04	0.79 ± 0.002	
						93	28 [26]	0.50 ± 0.003	33 ± 0.1	26 ± 0.1	1.3 ± 0.008	

a The solubility in SC is defined as *S*_SC_ = [*m*_AC-SC-sat_/(*m*_AC-SC-sat_ + *m*_SC_ + *m*_W-SC_) × 100%] (wt %) and the saturated mass fraction in SC is *F*_SC,sat_ = *m*_AC-SC-sat_/(*m*_AC-SC-sat_ + *m*_SC_) × 100% (wt %). The D_2_O water content in SC *W*_SC_ is defined as *m*_W-SC_/(*m*_SC_ + *m*_W-SC_) × 100% and was measured independently for each sample. The D_2_O water content was converted to equivalent H_2_O water content by assuming the same molar amount of water and shown in square brackets. *m*_AC-SC-sat_ and *m*_W-SC_ are the weights of the added chemicals AC and D_2_O water in SC, respectively, at the saturation condition of the added chemicals. *m*_SC_ refers to the dry weight of SC. Corresponding definitions of *S*_dSC_, *F*_dSC,sat_, and *W*_dSC_ are done for delipidized SC samples, where the subscript SC is replaced with dSC. D_2_O was used in all of these experiments. The physical properties including logarithm of octanol/water (H_2_O) partition coefficient (log *P*_O/W_), solubility of the chemicals in H_2_O *S*_W_ (g/L) at 32 °C, melting point *T*_m_ (°C), and molecular weight *M*_w_ (g/mol) are also shown. The added chemical/water D_2_O weight ratio in saturated solutions of the chemical *m*_AC-sat.sol._/*m*_W-sat.sol_ was calculated from *S*_W_ values assuming the same molar ratio of added molecules and water in H_2_O and in D_2_O.

b Measured as described in Supplementary Section 1.

c Results obtained from WAXS, while the remaining are from ^1^H NMR. The saturation concentration of caffeine in delipidized SC at RH = 93% was examined by WAXS (Figure S4) since the ^1^H signals of caffeine in delipidized SC at this condition are too low. The NMR data are presented as 95% confidence limit of the mean, whereas the range of the WAXS data is defined by the steps between the measured concentrations of the added chemical.

Delipidized SC was prepared from another batch of SC sheets (ca. 40 pig ears) according to previously published protocols.^21^ In short, the SC sheets were made into small pieces and the extracellular nonbound lipids were extracted by three solutions with different chloroform:methanol mixtures of ratios 2:1, 1:1, and 1:2 v/v. This sequence of extractions was performed at room temperature under gentle shaking for about 2 h. The remaining SC material was collected by filtration after each step. The whole extraction sequence with all three solvent mixtures was repeated for about 30 min for each extraction step. The filtered SC material was soaked in methanol overnight. In the final step, the methanol-extracted delipidized SC material was rinsed in Milli-Q water several times and dried under vacuum in a desiccator. During the extraction, the SC small pieces can be fragmented and after the extraction, we obtained a batch containing of very small grains of delipidized SC. The delipidized SC obtained from this procedure still contains covalently bound lipids in the cornified envelopes of the corneocytes.^15,21,22^ All experiments on delipidized SC were performed with samples from the same batch. It is noted that any lipid extraction protocol using apolar solvents may cause extraction of unwanted hydrophobic components and slightly polar compounds from the sample. Still, previous studies^6^ showed that soaking delipidized SC that was prepared with this protocol in water leads to further extraction of NMF, indicating that the preparation procedure using organic solvents does not remove all NMF from the sample. It is also pointed out that the molecular dynamics of the keratin protein and corneocyte envelope lipids are very similar for the corneocytes inside the SC sample and inside the delipidized SC prepared using the present protocol for a range of hydration conditions,^10^ implying that the extraction procedure does not change overall properties of the corneocytes significantly.

### 2.3 Preparation of NMR and WAXS Samples

Approximately 10–20 mg of dry pulverized SC or small grains of delipidized SC was added to an Eppendorf tube (Eppendorf, Germany) together with a controlled amount of added chemical and D_2_O. The samples and chemicals were added to the tube according to this order: the solid added chemical, then the SC (delipidized SC) powder, and finally D_2_O. Using this preparation procedure, almost all of the added D_2_O is first absorbed by the SC (delipidized SC) samples, and therefore the risk of the solid chemical being dissolved in D_2_O as a separate phase outside SC (delipidized SC) during the mixing is very low. The samples with known composition were then centrifuged, mixed, and equilibrated at 32 °C in closed chambers for 1 day with an RH of 93 and 97% (D_2_O), controlled with saturated solutions of KNO_3_ and K_2_SO_4_, respectively. The centrifugation step is to collect samples or chemicals that could otherwise stick to the walls of the Eppendorf tube and also helps the water absorption into SC (delipidized SC) samples as well as the mixing process to avoid the static in the powder. The water was never added in excess amount, and the RHs are always below 100%, meaning that there is no excess liquid water in the present samples. The final D_2_O water content of each individual SC or delipidized SC sample at a certain RH was determined by gravimetric measures before and after equilibration using a Sartorius R160D balance with a readability of 0.01 mg. Replicates of water content measurements therefore were performed for samples with the same composition that were measured by both NMR and WAXS. The samples were then transferred into tight rotor inserts to be fitted into a Bruker 4 mm rotor for NMR experiments or into a screw-tight sandwich cell, where the sample is contained between two polyimide film (Kapton) sheets for WAXS measurements.

The rationales of using pulverized SC (or delipidized SC) and adding a preliminary amount of water that is estimated to be close to the equilibrium water content were to reduce the equilibration time, as too long incubation times may cause problems with degradation of the biological SC sample. The preliminary amount of D_2_O added to the samples is almost the same as the D2O content determined from the gravimetric measures after 1 day for most samples, indicating that one has reached (or very close to) the equilibrium between water vapor phase in the RH chambers and water inside the samples. In previous studies,^7,20^ SC samples were prepared almost in the same way, i.e., mixing a certain amount of water and added chemicals with SC and then incubating the sample. No significant difference in ^13^C NMR signals and molecular mobility of both SC components and added chemicals between the samples incubated for 1 and 2 days was observed.^7^ One day is therefore considered long enough to reach equilibrium with respect to the water uptake. The absorption kinetics for the uptake of the added chemicals are expected to strongly depend on the grain size of the particles. One way to confirm that the sample is not in the absorption kinetic controlled limit is to vary the particle size. Here we studied the solubility of 2-MR in both pulverized SC and SC sheets after 1 day of incubation (Figure S1A), and the measured values were very similar. If the uptake was controlled by absorption kinetics, we would expect a large difference between the solubility in the samples with small grains and larger sheets. With respect to uptake of the added chemical, the absorption kinetics was studied for one sample (2% 2-MR in SC at 93%RH), showing that saturation was reached within 10 min (Figure S2), justifying that we reached equilibrium with respect to the added chemical for this sample after 24 h. For the remaining samples, we did not study adsorption kinetics, but still judge that 24 h equilibration is sufficient based on the consistency within the full set of NMR and WAXS data for all of the systems investigated (described in detail in the Results and Discussion section).

### 2.4 Experimental Reproducibility

In the present experimental approach, instead of performing several replicates for one sample prepared at one concentration, we studied a series of conditions (several samples with different concentrations of the added chemical) for each system. From the set of experiments performed for each chemical, we can confirm consistency within the whole experiment, in the NMR case, a linear increase until saturation is reached. In addition, we study each sample with two different experimental methods (NMR and WAXS), again to confirm internal consistency within the data sets for each system. The series of NMR data were analyzed through curve fitting, and from the fit of all data points we obtain the concentration at saturation. In case one of the data points is off, it would not strongly affect the overall outcome from the fit. The variability obtained from the fitting therefore includes variabilities of both sample preparation and the measurements, which are similar to the variability that would be obtained from replicated measurements of samples with the same composition. The biggest variability in the measurement of chemical uptake into SC is likely from the sample variation of the biological sample. This variability is minimized in this study by preparing a batch containing SC from several individuals, and all of the samples in one solubility measurement are from the same batch.

### 2.5 NMR Experiments

All ^1^H NMR experiments were performed on a Bruker Avance AVII-500 MHz NMR spectrometer (Bruker) with a Bruker 4 mm ^1^H/^13^C/^31^P HR (high resolution) MAS (magic-angle spinning) probe equipped with a MAS gradient coil and carried out at a spinning frequency of 5 kHz and at 32 °C. Free induction decays (FIDs) of ^1^H NMR measurements were recorded after a 10 μs 90° pulse using a spectral width of 20 ppm, an acquisition time of 0.05 s, 8 scans per experiment, a recycle delay of 20 s, and a receiver dead time of 4.5 μs. The FIDs were Fourier transformed with a line broadening of 10 Hz to obtain frequency domain NMR spectra. The ^1^H chemical shift of the terminal methyl peak at 0.9 ppm in SC and delipidized SC samples was used as an internal reference, while for the other samples, the calibration from previous experiments was kept. The following conditions were used to fit the data to find the value of *m*_AC-SC-sat_/*m*_SC_ corresponding to the saturated concentration
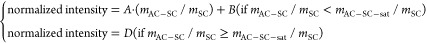
where *m*_AC-SC_ is the weight of the added chemical AC in the SC and *m*_SC_ is the dry weight of SC. The Monte Carlo error estimation method^23^ was used to estimate 1000 values of *m*_AC-SC-sat_/*m*_SC_ and its mean and 95% confidence intervals using a standard normal distribution of the sample. The same procedure was used to determine the saturation concentration in the delipidized SC (i.e., the subscript SC in the equations is replaced with dSC).

### 2.6 WAXS

WAXS experiments were performed using a SAXLab’s GANESHA 300 XL SAXS system (JJ X-ray, Denmark) equipped with a microfocus sealed tube X-ray source (GE’s Inspection Technology) and a 2D 300 K Pilatus Solid State detector (Dectris, Switzerland) at 32 °C. The temperature was controlled using a Julabo T Controller CF41 from Julabo Labortechnik GmbH (Germany). The scattering vector *q* is defined as *q* = (4π sin θ)/λ, where θ is half of the scattering angle and λ = 1.54 Å is the X-ray wavelength. Silver behenate which generates well-defined peaks in a *q* range of 0.1076 to 1.387 Å^–1^ was used as a reference to calibrate sample-to-detector distance and detector positions.^24^ The two-dimensional scattering pattern was radially averaged using SAXSGui software to obtain one-dimensional data without background correction.

## 3 Results and Discussion

We present a methodological strategy to distinguish molecules that are dissolved within a complex and heterogeneous material—here the SC—from molecules that are not incorporated but possibly deposited on the sample surface, and to determine the solubility of these compounds inside the SC. The method takes advantage of complementary information obtained from two different types of experiments, ^1^H MAS NMR^25^ and WAXS.^26^ We start by describing the results obtained for two model chemicals with different hydrophobicity, caffeine and 2-methyl resorcinol (2-MR). Caffeine is a cosmetics ingredient and is a standard model chemical used for skin absorption assays.^27^ 2-MR is a moderate skin sensitizer which can be found in hair dye formulations.^28^ We first measure the solubility of these chemicals in SC and in the delipidized SC at different water contents. Thereafter, we broaden the scope and present experimental data of solubility in SC for a range of other chemicals with different molecular structures and properties (Figure 2).

### 3.1 Combined NMR and SAXS Studies to Distinguish and Quantify Molecules Dissolved in SC

The strategy used to distinguish molecules that are dissolved in the SC from molecules that are not incorporated relies on the differences in the molecular properties of these molecules in the dissolved and excess conditions. Here, we have used chemicals that are present in a solid state in their pure form at ambient conditions. When these chemicals are taken up in the SC, they instead become dissolved. If we add an excess of solid chemicals, the fraction that is taken up in the SC is thus dissolved and present in a fluid environment, while the excess fraction remains solid (Figure 1B). We then use ^1^H NMR as a detection method, which only “sees” the dissolved mobile fraction of the chemical, and the excess solid fraction is invisible (Figure 1B,C). In the complementary WAXS experiments, which rely on that the added chemicals have crystalline structures, we take the opposite approach as we then only obtain diffraction patterns from the crystalline structures of the excess solid powder that has not been taken up in the SC (Figure 1B,C).

The experiments were performed on samples of SC that are mixed with varying amounts of a chemical of interest and D_2_O at 32 °C (Figure 1B). The samples were then equilibrated for 24 h at a controlled relative humidity (RH, using D_2_O) to leave time for the added chemical to dissolve in the SC matrix and the water content to adjust to a value corresponding to the chemical potential given by the RH of the environment. We then study these samples by means of ^1^H NMR and WAXS to detect the fraction of the added chemical that is dissolved in SC as well as the excess undissolved solid fraction. In the ^1^H NMR spectrum, solid components are observed as a very broad peak, whereas dissolved components that possess much faster molecular motion give rise to sharp peaks (Figure 1B).^25,29^ Here, the spectra were acquired at a spinning frequency of 5 kHz to improve the spectral resolution.^25^ WAXS, on the other hand, is sensitive to detect the nondissolved components that give rise to scattering pattern that originates from crystalline structures in the solid powder (Figure 1B).^26^ The NMR and WAXS experiments are performed for samples with varying amounts of the added chemical, and from the series of measurements it is possible to distinguish the saturation concentration of the chemical in SC. In the quantitative analysis, we integrate the sharp peaks from the added chemical in the ^1^H NMR spectra and plot the integrated values against the total amount of the chemical in the sample. Ideally, the saturation concentration as measured by the ^1^H NMR coincides with the concentration at which we see the appearance of sharp Bragg peaks in the WAXS spectrum (Figure 1C). From these combined experiments, we obtain a measure of the concentration of the chemical that is actually dissolved in the SC without interference of excess chemical that is deposited on the sample surface.

**Figure 3:**
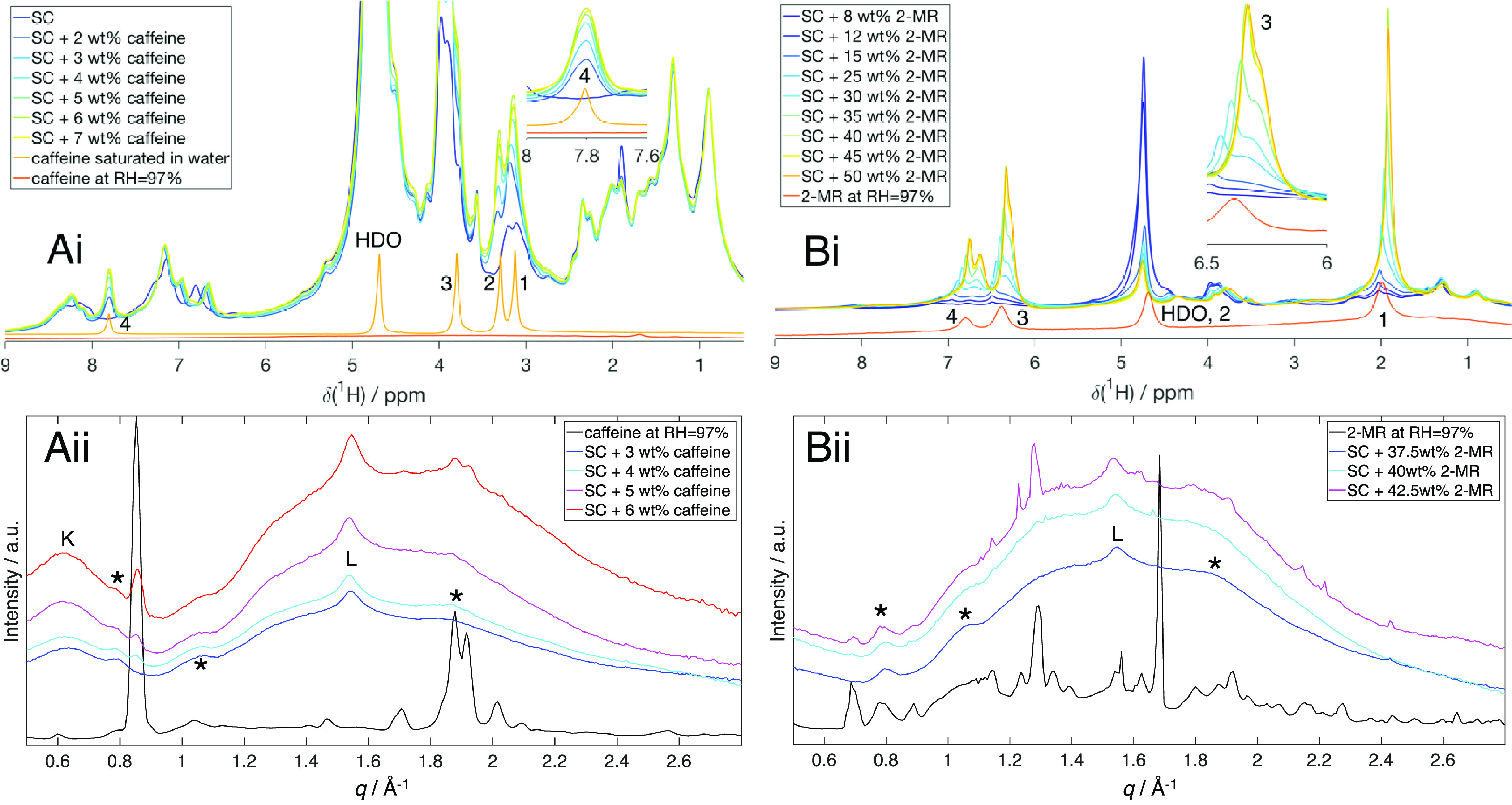
^1^H MAS NMR spectra (i) and WAXS profiles (ii) of SC with different amounts of caffeine (A) or 2-MR (B) at RH = 97% (D_2_O). The amount of the added chemicals in wt % refers to their mass fraction in SC calculated as *m*_AC-SC_/(*m*_AC-SC_ + *m*_SC_) × 100%. The NMR spectra of caffeine saturated in D_2_O (Ai) and at RH = 97% and of 2-MR at RH = 97% (Bi) and the WAXS profiles of caffeine (Aii) and of 2-MR (Bii) at RH = 97% are also shown. The HDO peak originates from residual protons in the D_2_O and exchanged protons from the SC. Scattering peaks from keratin interchain distance (K) and lipid chain packing (L) are also labeled; * indicates peaks from Kapton (see Figure S1B). The absence of keratin interchain distance peak in Bii is due to a large amount of dissolved 2-MR.

**Figure 4:**
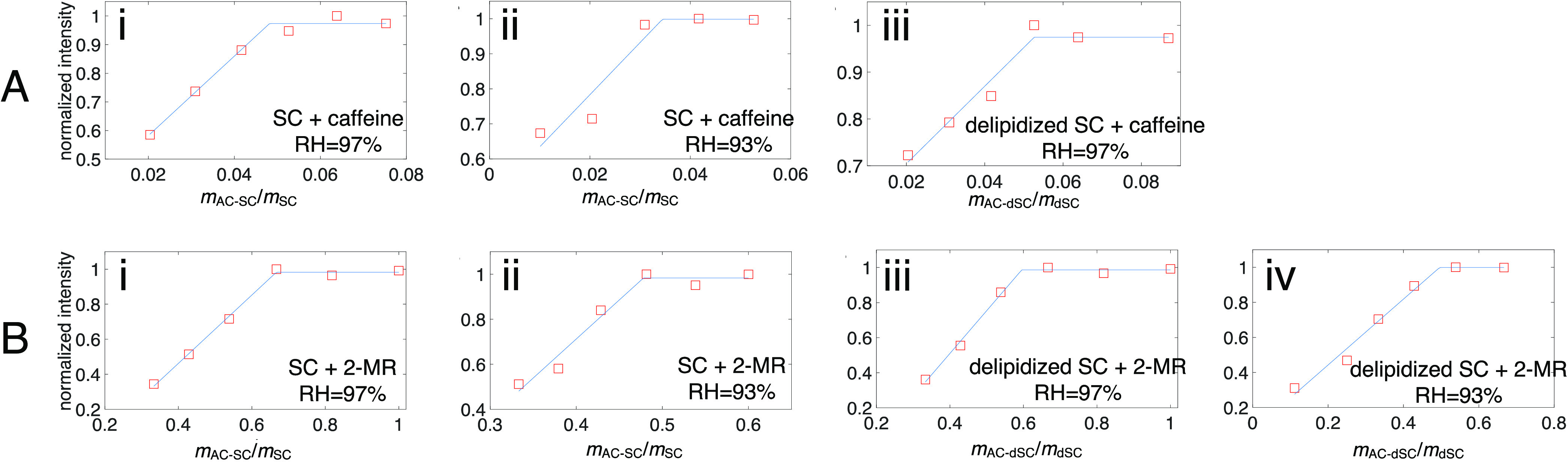
Normalized ^1^H peak intensity of hydrogen 4 of caffeine (A) or of hydrogen 3 of 2-MR (B) in SC or delipidized SC with different amounts of the added chemicals (AC), *m*_AC_/*m*_SC_ or *m*_AC_/*m*_dSC_, and at different RHs at 32 °C.

Figure 3A shows combined ^1^H NMR and WAXS data for caffeine in SC at 97% RH. Reference experiments were also performed for the caffeine alone and for SC without caffeine at the same conditions. The pure caffeine is solid at 97% RH, and it is therefore not observed in the ^1^H NMR spectra (Figure 3Ai). When caffeine is added to SC at the same RH, several sharp peaks coming from dissolved caffeine are distinguished in the ^1^H spectra. The intensities of these peaks increase with increasing concentration of caffeine until the saturation concentration (Figure 4Ai). In the present analysis, we focus on the peak of hydrogen 4 of caffeine (Figure 3Ai, inset) that is well resolved from SC resonances. The intensity of this peak does not increase when the total caffeine concentration exceeds 5 wt % in the SC sample. Linear regression fitting of the data then gives the saturation concentration in SC, *m*_AC-SC-sat_/*m*_SC_ (for more details, see Section 2.5). From the saturation concentration in SC, we obtain a measure of the solubility, here presented either as the mass fraction of the chemical with respect to the dry weight of the sample at saturation, *F*_SC,sat_, or as the solubility of the chemical in the hydrated SC, *S*_SC_. From these experiments, we can thus obtain a measure of the saturation concentration of caffeine in SC at any RH (Figure 4A, Table 1).

Next, the samples composed of SC and caffeine at 97% RH were examined using WAXS. Figure 3Aii shows the WAXS profiles of caffeine and its mixtures with SC at 97% RH. The solid powder of caffeine gives rise to multiple sharp WAXS peaks, which can be used as a signature to distinguish the conditions when we have excess solid caffeine in the samples, meaning total concentration above the saturation concentration. As seen in Figure 3Aii, we detect excess solid caffeine at *F*_SC_ = 4 wt % based on the WAXS peak at *q* = 0.85 Å^–1^. Other WAXS peaks originating from solid caffeine only appear at higher concentrations, and the intensities of all WAXS peaks increase with increasing amounts of excess (undissolved) caffeine. We therefore conclude that the saturation concentration of caffeine in SC at 97% RH obtained from WAXS is around 3–4 wt % which is close to the values obtained from the NMR measurements. From the combination of the ^1^H NMR and WAXS experiments, we are thus able to pinpoint the solubility of caffeine in the hydrated SC, and the data are summarized in Table 1.

### 3.2 Solubility of Chemicals with Different Hydrophobicity in SC and Delipidized SC at Different Hydration Conditions

The same approach was used to study the solubility of a more hydrophobic chemical, 2-MR, in SC. Figure 3Bi shows ^1^H NMR spectra obtained at RH = 97%. The different resonances of the neat chemical are detected as broad peaks at this RH. When dissolved in SC at the same RH, one can detect sharp peaks of 2-MR in SC. To analyze these data, we integrate the peak from hydrogen 3 of 2-MR (Figure 3Bi, inset) for varying amounts of 2-MR (Figure 4Bi). Although the excess of 2-MR can give rise to broad peaks as shown for the chemical alone at RH = 97% (Figure 3Bi), their intensities are negligible compared to those of the sharp peaks from the dissolved 2-MR. We therefore conclude that there is no further change in the signals from the dissolved 2-MR at a concentration above ca. 40 wt %. From the linear regression analysis, we get a measure of the saturation concentration of 2-MR in SC at 97% RH that is ca. 10 times higher than the saturation concentration of caffeine at the same RH (Table 1). The same samples were also examined using WAXS (Figure 3Bii). Here we only detect WAXS peaks corresponding to solid 2-MR at concentrations ≥40 wt % and no signs of solid 2-MR are seen at any lower concentrations (≤37.5 wt %).

One benefit of the presented methodology is that it enables studies of SC solubility at varying conditions, for example, at different RH conditions. The experiments with caffeine and 2-MR were performed for two different hydration conditions, RH = 97 and 93% (Figures 3, 4A-i-ii, 4B-i-ii, and S2). The saturation concentrations of both chemicals decrease with decreasing RH, both with respect to the total mass and the dry mass of the SC samples (Table 1). We also studied the solubility of the same chemicals in delipidized SC at the same RH conditions (Figures S3, S4, 4A-iii,4B-iii,iv, and Table 1), again showing the same trend in decreasing solubility with decreasing RH.

### 3.3 SC Solubility of Chemicals with Different Properties

Finally, the same analysis was performed for a range of other chemicals with different properties, including metronidazole, minoxidil, 7-ethoxycoumarin, and phenylethyl resorcinol at 97% RH (Table 2 and Figure S5). Metronidazole has antibacterial properties and is used in topical formulations for, e.g., the treatment of the chronic skin disease rosacea.^31^ Minoxidil is used to stimulate hair growth and treat androgenetic alopecia.^32^ 7-ethoxycoumarin belonging to a coumarin family that is widely used in cosmetic products is also used for metabolism characterization.^33^ And finally, phenylethyl resorcinol is a skincare and cosmetic ingredient due to its skin-lightening effect.^34^ As seen in Table 2, the lowest solubility (*F*_SC,sat_ < 2 wt %) is seen for metronidazole, minoxidil, and 7-ethoxycoumarin, whereas the highest solubility in SC is observed for phenylethyl resorcinol (*F*_SC,sat_ = 62–65 wt %). The solubilities of the different chemicals in SC at the same 97% RH (Tables 1 and 2) show that for these chemicals neither the tabulated octanol/water partition coefficient log *P*_O/W_, nor the water solubility, nor the molecule size is sufficient to explain the observed variations in SC solubility. We finally point out that the precision and resolution of the present methodology can be improved by including more measurement points.

**Table 2:** Solubility of Metronidazole, Minoxidil, 7-Ethoxycoumarin, and Phenylethyl Resorcinol in SC at RH = 97% Obtained from WAXS and Their Physical Properties^a^

chemical	log *P*_O/W_	*S*_w_	*T*_m_	*M*_w_	RH	*W*_SC_	*F*_SC,sat_	*S*_SC_
metronidazole	–0.02^30^	11^30^	160	171	97	50	<2	<1.0
minoxidil	1.24^30^	2.2^30^	248^30^	209	97	50	<2	<1.0
7-ethoxycoumarin	2.3^30^	0.81^35^	88–90	190	97	50	<2	<1.0
phenylethyl resorcinol	3.5^30^	0.26^35^	78–79^36^	214	97	50	62–65	44–48

a The same notations as in Table 1 are used. D_2_O was used in all of these experiments, the only exception is the solubility in water *S*_w_ where H_2_O was used. The range of the presented data is defined by the steps between the measured concentrations of the added chemical.

### 3.4 Method to Distinguish and Quantify Molecules That Are Taken Up into a Complex Biomaterial from Molecules That Are Present in Excess

The present methodology makes it possible to distinguish between the fraction of the added compound that is actually dissolved inside the SC and an excess fraction of the same compound that is not taken up in the SC but may still be deposited on its surface or in furrows. This methodology is based on the detection methods that act as a dynamic filter to spot only one of the fractions either the dissolved fraction in SC (^1^H NMR) or the excess solid one (WAXS). There are numerous other methods to study solubility in SC presented in the literature. Many of these approaches use SC that is fully hydrated or at an excess volume of a relatively dilute aqueous solution.^3−5,37,38^ The methodology presented here provides an important complement to these previous studies as it is applicable for a large range of hydration conditions, also low RHs. In addition, it avoids the risks of extraction of SC components—in particular NMF and shorter-chain lipids^6,7,18,19^—to the solution that is in contact with the SC. Another advantage of the present method is that it is possible to study the uptake of hydrophobic chemicals, good candidates for skin permeation,^39,40^ without the need to use organic solvents that also influence the mobility of the SC lipid and protein components.^7,41−43^ Finally, we point out that one can use the same experimental approach as used here for equilibrated samples for time-resolved (minute timescale) studies of uptake of different chemicals into SC, as exemplified for 2-MR in Figure S2.

One requirement for the present methodology is that the pure chemical is present in its solid form at the investigated hydration and temperature. If the compound is dissolved in the excess water that is outside SC, this would interfere with the measurement since it is then not possible to distinguish between the chemical that is dissolved in SC and that dissolved in any excess solution. For the latter cases, the solubility in SC can still be estimated if one also knows the solubility of the chemical in the solvent as well as the total volume of the excess solution. The added chemical should also be compatible with the detection methods. The use of ^1^H MAS NMR requires resolved resonances of the added chemicals in the ^1^H NMR spectrum, whereas WAXS is only applicable for crystalline solid chemicals. The methodology to have a measurement that only “sees” one of the fractions—excess or dissolved—is general and could in principle be translated also to other experimental methods. For example, higher-resolution ^13^C INEPT (insensitive nuclei enhanced by polarization transfer) NMR experiments^44^ only show the ^13^C signals of the dissolved fraction of the added chemical with C-H bond rotational correlation time faster than ca. 10 ns.^45^ One can also use the more time-consuming *Q* (quantitative)-INEPT method^13^ to quantify the amount of solubilized added chemical compared with the amount of SC mobile components in a sample with an excess amount of added solid chemical. Another possible approach is fluorescence spectroscopy using a fluorophore of whose spectra can be shifted when changing its local environment.^46^ Finally, it is noted that the detection limit of these approaches can vary between chemicals due to differences in the NMR and WAXS spectra of the pure chemicals in combination with SC (or delipidized SC) as well as differences in instrumental setups with different measurement times and/or beam intensities. We also note that both NMR and WAXS spectra differ between SC and delipidized SC, meaning that the detection limit where signals from the added compound can be resolved may vary between the different types of samples.

### 3.5 Solubility and Lipid/Corneocyte Distribution

The uptake of chemicals into SC from a formulation is often predicted using values of octanol/water partition coefficient log *P*_O/W_ of the chemicals together with their solubility in the formulation of interest (reviewed in refs (3, 47)). In the current study, the measured solubilities of six different chemicals in SC at the same 97% RH are shown in Tables 1 and 2. It is clear that for these chemicals neither log *P*_O/W_, nor water solubility, nor molecule size is sufficient to explain the observed variations in SC solubility. The comparison thus illustrates the complications of capturing the important features of the responding SC biological material by simple nonresponding solvent systems like octanol and water. One example of this is the response in SC to changes in skin hydration.^10,15^ From the presented data (Table 1), it is clear that the saturation concentration of the added chemicals in SC depends on the water chemical potential (related to water content) in the sample. It is here important to point out that the properties of the SC molecular components are not constant but can be altered by external conditions, for example, the SC hydration^10,12,15,48^ and interactions with added molecules.^7,20,41,49−52^ A closer inspection reveals that the proportions between the saturation amount of the added chemicals and the water content can vary with the RH conditions (Table 1). The solubility will thus not only depend on the water content but also on the properties of SC components, where one key aspect is the proportion of fluid lipids in the extracellular matrix^13^ and the swelling of the different SC regions in water.^10^ The increase in the RH of the skin surrounding will not only change the water content in SC (Table 1)^10,12,14^ but also lead to fluidization of a small portion of the lipids and an increased mobility in the terminal amino acids of the keratin filaments inside the corneocytes.^9,10,15^ One can therefore expect increased solubility of any added chemicals in SC when the hydration increases, which is also the case for the model compounds investigated here (Table 1). It is also possible that the dissolution of any added chemical inside the SC will lead to a shift in the solid–fluid balance toward higher content of fluid components. This may also lead to changes in the mobility of added chemicals in SC.^7,53^

The present methodology can be extended to also determine the distribution of an added chemical between different regions of a complex biomaterial by measuring the solubility in each of the regions in conditions that correspond to the same equilibrium. With respect to the present system, it is possible to extract the distribution of an added compound between the corneocytes and the extracellular lipids within the SC. This requires separate measures of the solubility experiments for SC, delipidized SC, and extracellular lipids in conditions where the chemical potentials of the different components are the same for all experiments. As outlined in Figure 1D, the solubility of the added chemicals can be measured for the different samples (SC, delipidized SC, and lipids, preferably from the same batch of SC) at saturation conditions, as the chemical potential of the compound is then controlled and given by the chemical potential of the pure compound. With respect to the water, its concentration will vary between the extracellular lipid regions and the corneocytes inside SC. However, if the samples are all equilibrated at the same RH (Δμ_w_ = *RT *ln RH), the water chemical potential, Δμ_w_, is the same in all parts of the SC sample (*R* is the gas constant and *T* is the temperature).

When evaluating data of solubility in SC and its lipid and corneocytes regions, it is important to bear in mind the SC lipid fraction of fluid SC lipids is minor compared to the fraction of solid SC lipids,^13^ and the uptake of the chemical will naturally vary between the co-existing solid and fluid lipid domains. In general, the solubility of any chemical is expected to be much higher in a fluid matrix compared to a solid. The average solubility in the SC lipids therefore will be much lower compared to the solubility in fluid lipids only, which would be more similar to the octanol solution. In addition, the SC lipids form self-assembled structures with apolar and polar domains within the lamellar arrangement, which cannot be captured by a simple octanol solution. This will be relevant in particular to the dissolution of amphiphilic and polar molecules in the SC lipid domains. Turning to the delipidized SC, the measured proportions of the added compounds and water inside these samples may exceed the saturation concentration in a water solution (Table 1). This finding indicates that the solubilization in the delipidized SC is also affected by interactions with other components besides water, which was also suggested in ref (37). The corneocytes can be seen as water-rich regions filled with keratin filaments, containing both hydrophilic and hydrophobic amino acids.^54^ Previous studies^7,53^ also showed that chemicals can be taken up by corneocytes even under dry condition without water.

### 3.6 Quantitative Comparisons with Previous Studies

Most of previous studies on solubility in SC report on the partition coefficients of added chemicals between SC and an excess aqueous solutions^4,55^ rather than the maximum amount of the added chemical dissolved within SC at a given hydration. This implies that one cannot make direct quantitative comparisons between the present data and literature data, but still, we can comment on overall trends on similarities and differences. Based on the reported values of partition coefficients and solubility in water, we can make estimates of the solubilities of added chemicals in SC at full hydration (Supplementary Section 2 and Table S1). For caffeine, the solubility in porcine and human SC at full hydration was estimated from literature data^4,55^ to be 2.8–4.3 wt % (using D_2_O), which is higher but still in the same range as the herein presented value of 2.4 wt % at RH 97% (using D_2_O, Table 1). As discussed above, the solubility in SC is expected to increase with increasing hydration. Even though the humidity drop from 100% to 97% may appear small, it can have a large impact at the molecular level. It is clear from the sorption isotherms of SC that the slope in terms of water uptake per unit change in RH is very steep in the higher RH range, meaning that there is a clear difference in water content in the SC samples at the different relative humidity conditions.^10^ The observed differences are thus in line with the previous reported data. It is also noted that the reported values of partition coefficients were obtained from the experiments where the chemicals were added together with organic solvents,^4,55^ which may also influence the SC fluidity^7,52^ and thereby also the SC ability to dissolve the added chemicals.

## 4 Conclusions

Based on the here presented methodology, one can measure the solubility in SC and delipidized SC, and one may also obtain estimate of the distribution of the added chemical between the extracellular lipids and the corneocytes at any hydration condition. The increase in solubility in SC and the redistribution of the added chemicals between the different lipid and corneocyte regions at varying hydration conditions can have a large impact on SC permeability. These variations can also impact the dermal chemical safety and efficiency assessment if not performed at relevant hydration conditions. The experiments further demonstrate that rather large amounts of solid chemicals can actually be dissolved in the SC on a minute timescale, pointing at the potential risks related to accidental exposure to such solid chemicals. This is also in line with previous studies^56^ using Franz diffusion cell. In this case, the chemical is noted to be present at a saturated concentration or an activity of unity, corresponding to the highest driving force for skin penetration. The same approach to measure solubility can be applied to other systems with low water content. Other applications may be found in dry formulations in food, drug delivery, and cosmetics, where the solubility of constituents in the formulation changes during freeze-drying or exposure to dry air, resulting in segregation or precipitation.

## Supporting Information Available

WAXS profiles of SC sheets and 2-MR (A) and of Kapton only or SC and caffeine (B) (Figure S1); WAXS profiles of SC and 2-MR recorded during different time points after mixing (Figure S2); ^1^H NMR data of SC or delipidized SC and caffeine or 2-MR (Figure S3); WAXS profiles of delipidized SC and caffeine (Figure S4); WAXS profiles of SC and other added chemicals (Figure S5); ^1^H NMR data of saturated solutions of caffeine and 2-MR (Figure S6); estimated solubility of caffeine and 7-ethoxycoumarin in SC at full hydration (Table S1); measuring solubility of caffeine and 2-MR in H_2_O (Section 1); and calculations of solubility of added chemicals in SC at fully hydrated condition (Section 2) (PDF)
