# Supplementary material for: Solubility of Foreign Molecules in Stratum Corneum Brick and Mortar Structure
Source: Langmuir. 2023 Jan 30;39(6):2347–57. doi: 10.1021/acs.langmuir.2c03092 (PMC9933541; doi:10.1021/acs.langmuir.2c03092)
Supplement: Supplementary file 1 — la2c03092_si_001.pdf [file la2c03092_si_001.pdf]

Supplementary information

Solubility of foreign molecules in stratum corneum brick and mortar structure

Quoc Dat Pham,[a,b*] Bruno Biatry,[c] Sébastien Grégoire,[c] Daniel Topgaard,[a] Emma Sparr[a]

[a] Division of Physical Chemistry, Chemistry Department, Lund University, P.O. Box 124, 22100 Lund, Sweden

[b] Gillette Reading Innovation Centre, 460 Basingstoke Road, Reading, RG2 0QE, Berkshire, UK

[c] L'Oréal Research & Innovation, 1, avenue Eugène Schueller, 93601 Aulnay sous Bois, France

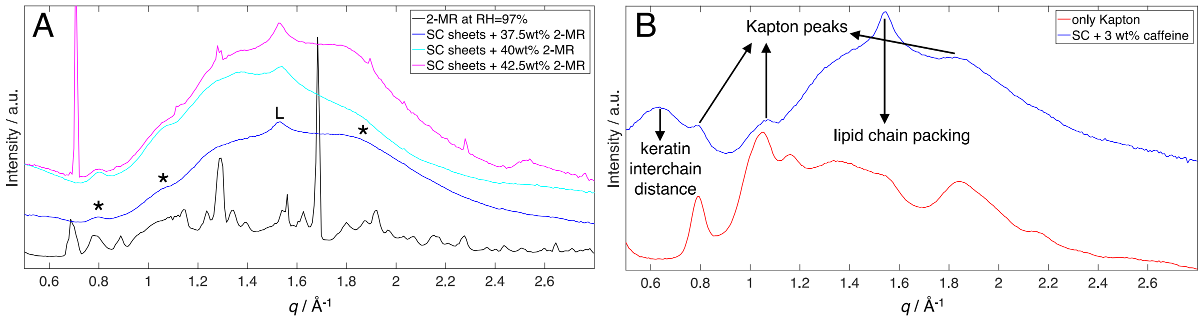

Fig. S1. (A) WAXS profiles of 2-MR and of SC sheets with different amount of 2-MR at RH=97% (D_2O) and at 32 °C. The sample was prepared and equilibrated in the same way as pulverized SC samples. The data show that the saturated mass fraction in SC $F_{SC,sat}$ at 97% RH of 2-MR in SC sheets is 40-42.5 wt% which is rather similar to the data obtained for pulverized SC (Table 1). Scattering peaks from keratin interchain distance (K) and lipid chain packing (L) are also labeled. * indicates peaks from Kapton (see Fig. S1B). (B) WAXS profiles of only Kapton and of pulverized SC with 3 wt% caffeine at 97% RH. The amount of the added chemicals in wt% refers to their mass fraction in SC calculated as $m_{AC\text{-}SC}/(m_{AC\text{-}SC} + m_{SC})\cdot 100\%$. $m_{AC\text{-}SC}$ and m_{SC} are the weight of the added chemicals AC in SC and the dry weight of SC, respectively.

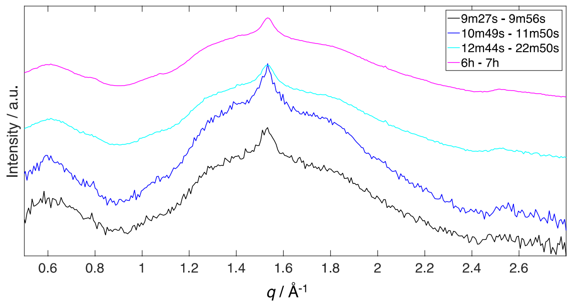

Fig. S2. WAXS profiles recorded during different time points after mixing SC and pure 2-methyl resorcinol (with a proportion corresponding to 2 wt% 2-methyl resorcinol in samples of SC and 2-methyl resorcinol) that had been equilibrated separately at RH=93% (D_2O) and at 32 °C. The earliest measured time point is limited by the instrument operation. After 9.5 minutes after mixing, we could not detect any signs of solid 2-MR in the sample, indicating that the dissolution of solid 2-MR into SC at RH=93% is faster than 9.5 minutes. The time resolution can be improved by using synchrotron WAXS instruments with higher flux together with experimental set-ups where the time gap between mixing and starting the measurement is shorter. The amount of the added chemicals in wt% refers to their mass fraction in SC calculated as $m_{AC\text{-}SC}/(m_{AC\text{-}SC} + m_{SC})\cdot 100\%$.

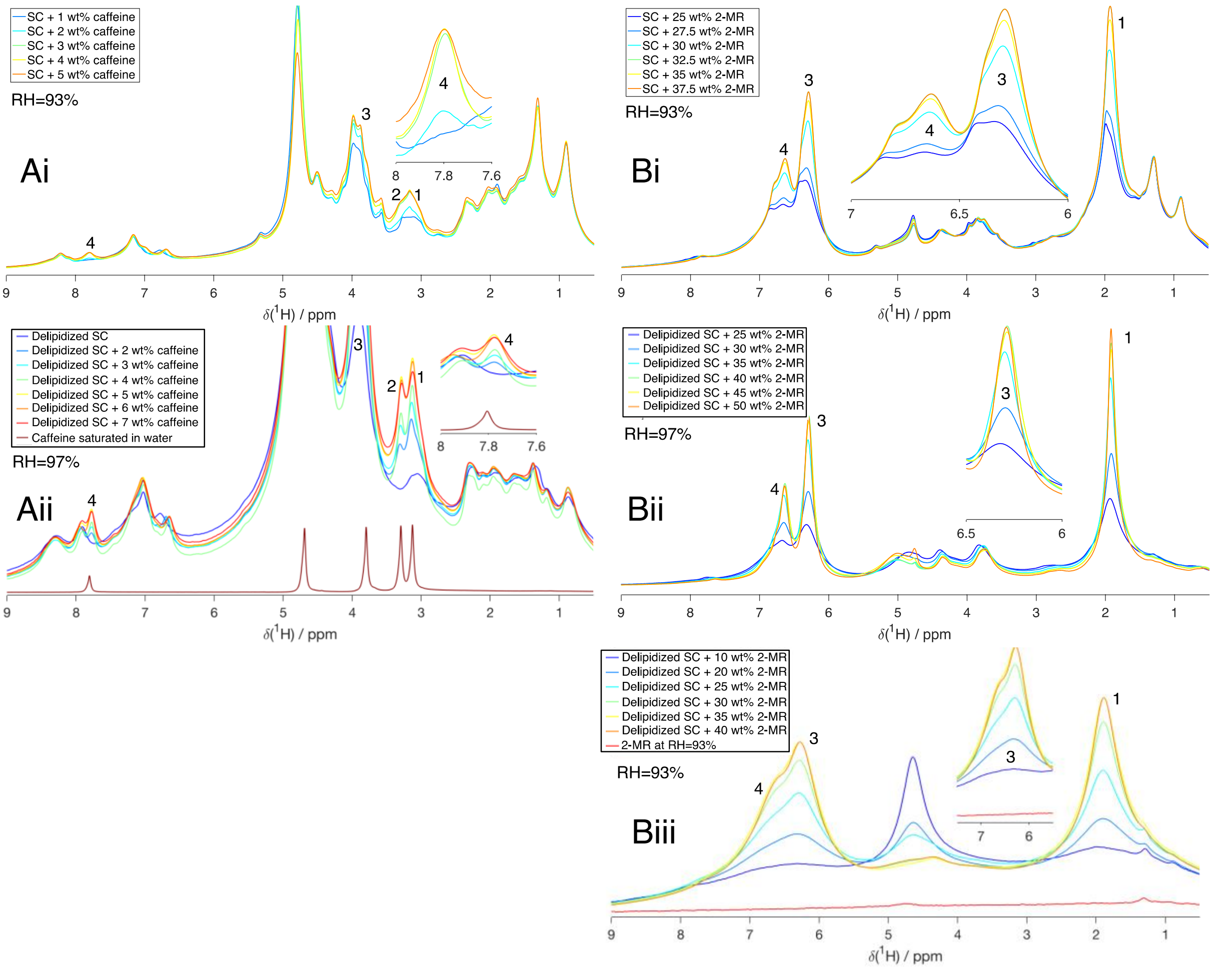

Fig. S3. ^{1}H MAS NMR spectra of SC (i) and delipidized SC (ii-iii) with different amount of caffeine (A) or 2-methyl resorcinol (2-MR) (B) at different RHs (D_2O) and at 32 °C. The amount of the added chemicals in wt% refers to their mass fraction in SC (delipidized SC) calculated as $m_{AC\text{-}SC(dSC)}/(m_{AC\text{-}SC(dSC)} + m_{SC(dSC)})\cdot100\%$.

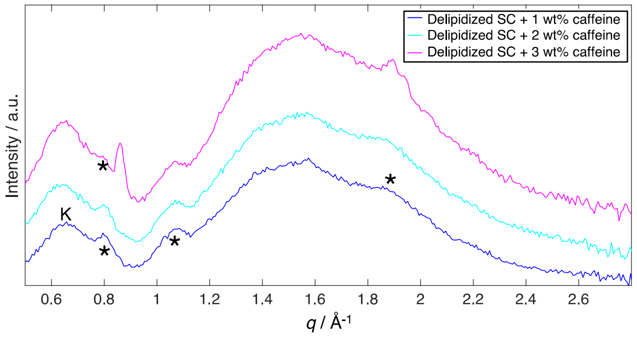

Fig. S4. WAXS profiles of delipidized SC with different amount of caffeine at RH=93% (D_2O) and at 32 °C. WAXS peak of lipid chain packing disappears in the profile of delipidized SC, indicating the extraction of lipids. Scattering peaks from keratin interchain distance (K) and Kapton (*) are also labeled. The amount of the added chemicals in wt% refers to their mass fraction in delipidized SC calculated as $m_{AC\text{-}dSC}/(m_{AC\text{-}dSC} + m_{dSC})\cdot100\%$.

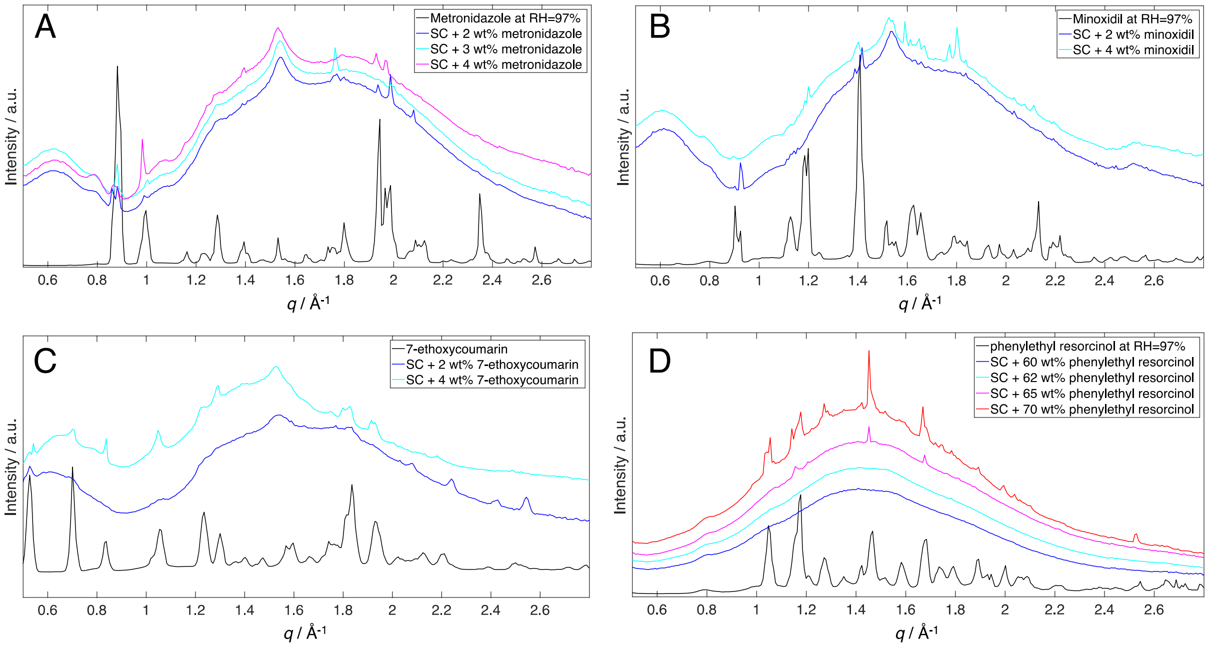

Fig. S5. WAXS profiles of added chemicals and of SC with different amount of added chemical at RH=97% (D_2O) and at 32 °C: metronidazole (A), minoxidil (B), 7-ethoxy coumarin (C), and phenylethyl resorcinol (D). The amount of the added chemicals in wt% refers to their mass fraction in SC calculated as $m_{AC\text{-}SC}/(m_{AC\text{-}SC} + m_{SC})\cdot 100\%$.

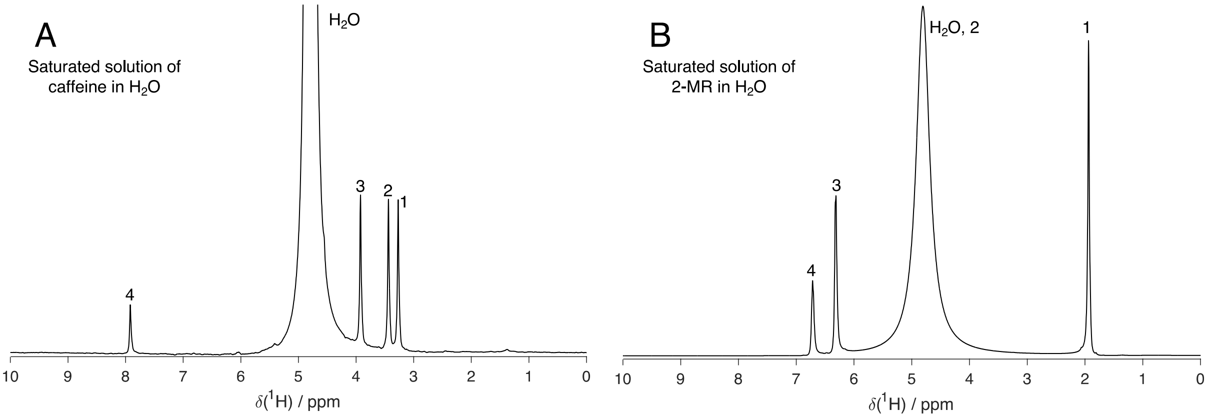

Fig. S6. ^1H MAS NMR spectra of saturated solution of caffeine (A) and 2-MR (B) in H_2O at 32 °C.

Supplementary Section 1. Measuring solubility of caffeine and 2-MR in H_2O

The solubility of the added chemicals in H_2O was measured on samples of saturated chemical in H_2O equilibrated with excess amount of the solid chemical at 32 °C for 2 weeks. From the area ratio of the peaks in ^1H MAS NMR spectra of the saturated solutions at 32 °C (Fig. S6), one can estimate the molar ratios of the chemical and H_2O of 407 mol H_2O per mol caffeine and 14.6 mol H_2O per mol 2-MR, corresponding to 26 g caffeine per 1000 g caffeine and H_2O and 321 g 2-MR per 1000 g 2-MR and H_2O. If we assume the density of the saturated solution of the chemicals is 1000 g/L, we can obtain the solubility in water of caffeine of 26 g/L and of 2-MR of 321 g/L.

Supplementary Section 2. Details on calculations of solubility of added chemicals in SC at fully hydrated condition from previous studies[1, 2]

Most of previous studies on solubility in SC report on the partition coefficients of added chemicals between SC and an excess aqueous solutions.[1, 2] We need to recalculate the maximum amount of the added chemical dissolved within SC from the partition coefficients in order to make direct quantitative comparisons with the data in Table 1. The partition coefficient $K_{\mathrm{SC/W}}$ of chemicals between SC and an aqueous solution (W) is defined as the ratio between the solubility in SC and the solubility in water, expressed in mass of the chemical per volume of SC or the aqueous solution. From the literature data on measured partition coefficients and solubility of the chemical in water, we can calculate the maximum amount of the added chemical dissolved within SC per volume of SC. This can then be recalculated to S_{SC} by knowing the density of fully hydrated SC.

$K_{\mathrm{SC/W}}$ is defined as

$$K_{\mathrm{SC/W}} = \frac{\left(\frac{m_{\mathrm{AC-SC}}}{V_{\mathrm{fully\ hydrated\ SC}}}\right)}{\left(\frac{m_{\mathrm{AC-W}}}{V_{\mathrm{W}}}\right)} \quad \text{(S1)}$$

where $m_{\mathrm{AC\text{-}SC}}$ and $m_{\mathrm{AC\text{-}W}}$ refer to the weight of the added chemical (AC) in SC and in the aqueous solution (W), respectively. $V_{\mathrm{fully\ hydrated\ SC}}$ and V_{W} refer to the volume of fully hydrated SC and volume of the aqueous solution. In saturated condition of chemical, $\frac{m_{\mathrm{AC-W}}}{V_{\mathrm{W}}}$ corresponds to the solubility of chemical in water S_{W} (mg/mL).

The volume of hydrated SC has been proposed to be 3.518 mL per 1 g dry human SC[3] or

$$V_{\mathrm{fully\ hydrated\ SC}} = 3.518 \cdot 10^{-3}\, m_{\mathrm{SC}} \quad \text{(S2)}$$

in which the units of $V_{\mathrm{fully\ hydrated\ SC}}$ and m_{SC} are mL and mg, respectively.

From Eq. S1 and S2, we then obtain the following expression:

$$K_{\mathrm{SC/W}} = \frac{m_{\mathrm{AC-SC-sat}}}{3.518 \cdot 10^{-3} m_{\mathrm{SC}} S_{\mathrm{W}}} \quad \text{(S3)}$$

$$\frac{m_{\mathrm{AC-SC-sat}}}{m_{\mathrm{SC}}} = 3.518 \cdot 10^{-3} S_{\mathrm{W}} K_{\mathrm{SC/W}} \quad \text{(S4)}$$

in which $m_{\mathrm{AC\text{-}SC\text{-}sat}}$ is the saturated weight of the added chemical (AC) in SC.

The saturated mass fraction of chemical in SC $F_{\mathrm{SC,sat}}$ at fully hydrated condition is then calculated as

$$F_{\mathrm{SC,sat}} = \frac{m_{\mathrm{AC-SC-sat}}}{m_{\mathrm{AC-SC}} + m_{\mathrm{SC}}} = \frac{3.518 \cdot 10^{-3} S_{\mathrm{W}} K_{\mathrm{SC/W}}}{1 + 3.518 \cdot 10^{-3} S_{\mathrm{W}} K_{\mathrm{SC/W}}} \quad \text{(S5)}$$

Using the solubility in H_2O water of caffeine (26 mg/mL, Table 1) and $K_{\mathrm{SC/W}}$ value for caffeine obtained from pig SC[1] or human SC[2] in previous studies, we can obtain $F_{\mathrm{SC,sat}}$ values of caffeine in fully hydrated condition according to Eq. S5 and the values are shown in Table S1. The value of $K_{\mathrm{SC/W}}$ can also be estimated from log $P_{\mathrm{O/W}}$ (reviewed in [4]) but we herein use experimental values for this estimation. Using a water content of 2.75 g H_2O water per 1 g dry SC (or 3.05 g D_2O water per 1 g dry SC) for fully hydrated human SC,[3] the solubility of caffeine in SC at full hydration then can be estimated in Table S1. It is noted that the volume of hydrated SC and the water content per 1 g dry SC used in the estimation are of human SC.

Table S1. Estimated solubility of caffeine in SC at full hydration from the partition coefficients $K_{SC/W}$ obtained from previous studies.[1, 2] The solubility in SC is defined as S_{SC}=[$m_{AC\text{-}SC\text{-}sat}$/($m_{AC\text{-}SC\text{-}sat}$+ m_{SC}+ $m_{W\text{-}SC}$) · 100%] (wt%) and the saturated mass fraction in SC is $F_{SC,sat}$=$m_{AC\text{-}SC\text{-}sat}$/($m_{AC\text{-}SC\text{-}sat}$+ m_{SC}) · 100% (wt%). $m_{AC\text{-}SC\text{-}sat}$ and $m_{W\text{-}SC}$ are the weights of the added chemicals and water in SC, respectively at the saturation condition of the added chemical. m_{SC} refers to the dry weight of SC. Solubility of the chemicals in H_2O water S_W (mg/mL) is also shown.

	S_W	$K_{SC/W}$	$m_{AC\text{-}SC}/m_{SC}$	$F_{SC,sat}$	S_{SC} in H_2O	S_{SC} in D_2O
**Caffeine**	26	1.27 [1]	0.12	10.4	3.0	2.8
		2.0 [2]	0.18	15.5	4.7	4.3

References

(1) Rothe, H.; Obringer, C.; Manwaring, J.; Avci, C.; Wargniez, W.; Eilstein, J.; Hewitt, N.; Cubberley, R.; Duplan, H.; Lange, D.; et al. Comparison of protocols measuring diffusion and partition coefficients in the stratum corneum. *J. Appl. Toxicol.* **2017**, *37* (7), 806-816.
(2) Ellison, C. A.; Tankersley, K. O.; Obringer, C. M.; Carr, G. J.; Manwaring, J.; Rothe, H.; Duplan, H.; Géniès, C.; Grégoire, S.; Hewitt, N. J.; et al. Partition coefficient and diffusion coefficient determinations of 50 compounds in human intact skin, isolated skin layers and isolated stratum corneum lipids. *Toxicol. In Vitro* **2020**, *69*, 104990. DOI: https://doi.org/10.1016/j.tiv.2020.104990.
(3) Nitsche, J. M.; Wang, T.-F.; Kasting, G. B. A Two-Phase Analysis of Solute Partitioning into the Stratum Corneum. *J. Pharm. Sci.* **2006**, *95* (3), 649-666. DOI: 10.1002/jps.20549 (acccessed 2019/08/08).
(4) Mitragotri, S.; Anissimov, Y. G.; Bunge, A. L.; Frasch, H. F.; Guy, R. H.; Hadgraft, J.; Kasting, G. B.; Lane, M. E.; Roberts, M. S. Mathematical models of skin permeability: an overview. *Int J Pharm* **2011**, *418* (1), 115-129. DOI: 10.1016/j.ijpharm.2011.02.023.
